# Effect of dietary inclusions of bitter kola seed on geotactic behavior and oxidative stress markers in *Drosophila melanogaster*

**DOI:** 10.1002/fsn3.782

**Published:** 2018-10-26

**Authors:** Ganiyu Oboh, Opeyemi B. Ogunsuyi, Mopelola T. Ojelade, Seun F. Akomolafe

**Affiliations:** ^1^ Department of Biochemistry Federal University of Technology Akure Nigeria; ^2^ Department of Biomedical Technology Federal University of Technology Akure Nigeria; ^3^ Department of Biochemistry Ekiti State University Ado-Ekiti Nigeria

**Keywords:** antioxidant, *Drosophila melanogaster*, *Garcinia kola* seed, saponin toxicity

## Abstract

This study evaluated the effect of dietary inclusions of *Garcinia kola* (GK) seed on geotactic behavior and some oxidative stress markers in wildߚtype fruit flies (*Drosophila melanogaster*). Flies were raised on diet supplement with GK seed for 5 days. The negative geotactic behavior of flies which was used to evaluate their locomotor performance was thereafter evaluated. The flies were subsequently homogenized and the reactive oxygen species (ROS) level, acetylcholinesterase (AChE), catalase and glutathioneߚSߚtransferase (GST) activities, as well as nitric oxide (NO) and total thiol contents were assayed. The phytochemical constituents of GK seed were also determined. It was observed that higher dietary inclusions of GK seed reduced the survival rate of *D. melanogaster* more significantly compared to control flies. Also, higher dietary inclusions of GK seed significantly reduced locomotor performance and AChE activity, while the ROS level was increased compared to the control. Activities of GST and catalase were significantly increased in flies fed diet supplemented with higher GK seed inclusions but their NO content was significantly reduced compared to control. Phytochemical analysis of GK seed revealed abundance of saponin > glycosides > alkaloids > phenols > flavonoids. These results have shown that dietary inclusion of GK seed at higher concentrations reduced survival rate of *D. melanogaster* and impaired cholinergic system, with elevated activities of some antioxidant enzymes under acute exposure. These observations could be associated with bioactivities of predominant phytochemicals in GK seed such as saponin and glycosides which have been reportedly toxic at high concentration. Therefore, this study suggests that high consumption of GK seed could induce some toxicological effects and moderate consumption is hence recommended.

## 1 INTRODUCTION

Edible plant seeds have been in existence for many years, Bitter kola *(Garcinia kola)* is one of the edible plant seeds that are well consumed in Nigeria and other parts of sub‐Saharan Africa. The seeds of bitter kola are highly valued for its stimulating effects (Atawodi, Mende, Pfundstein, Preussmann, & Spiegelhalder, 1995). Bitter kola seed is used broadly in African folklore as therapeutic interventions for diverse diseases (Farombi, Akanni, & Emerole, 2002; Onunkwo, Egeonu, Adikwu, Ojile, & Alowosulu, 2004), such as for treatment of cough, as antipurgative and as aphrodisiac (Madubunyi, 1995; Okunji & Iwu, 1991). Studies showed that predominant phytochemicals in bitter kola seed includes saponin, tannin, and oxalates among others.

Reactive oxygen species (ROS) are harmful molecules, capable of inducing oxidative damage in amino acids, proteins, lipids, and DNA components of tissues at higher levels (Sharma, 2015; Valko, Rhodes, Moncol, Izakovic, & Mazur, 2006). The two main lines of defense against oxidative stress are endogenous antioxidant molecules (Abolaji, Kamdem, & Lugokenski, 2015) and repair enzymes that remove or repair oxidatively damaged macromolecules. Also, living organisms can withstand damages caused by oxidative stress in reaction to pro‐oxidants through the process of adaptive response (Crawford & Davies, 1994). It has been reported that adaptive response exists in prokaryotes (Farr, Natvig, & Kogoma, 1985; Greenberg & Demple, 1989; Hassan & Fridovich, 1977) and eukaryotes (Laval, 1988; Lu, Maulik, Moraru, Kreutzer, & Das, 1993; Spitz, Dewey, & Li, 1987) exposed to various oxidative stress conditions.

Fruit fly (*Drosophila melanogaster*) is an arthropod, belonging to the family Drosophillidae. *D. melanogaster* has become a model for biological research since its introduction over 100 years ago, especially in genetics and molecular biology (Abolaji, Kamdem, Farombi, & Rocha, 2013). *Drosophila* is highly sensitivity to varying degrees of toxicants and is considered as a model for toxicity studies (Abolaji et al., 2013). It also serves as a useful model for evaluating biological actions of therapeutic agents against several human diseases (Adedara, Klimaczewski, 2015; Adedara, Rosemberg, 2015). *Drosophila* has unique physiological features similar to that of vertebrates (Baker & Thummel, 2007), and it has been certified to help reduce, refine, and replace higher animal usages such as rodents in biomedical research such as toxicity studies (Benford et al., 2000).

The awareness that medicinal plants can play a major role in ameliorating disease conditions is often overshadowed by the dosage intake of these medicinal plants. *Garcinia kola* (GK) seeds have long been consumed among various cultures of sub‐Saharan Africa for various purposes including recreational and tradio‐medicinal purposes. It is often consumed among various cultures of Nigeria especially for its stimulatory effects (Atawodi et al., 1995). Although previous studies have reported various extracts and fractions of GK seed for their therapeutic uses in treating throat infections, bronchitis, cough, hepatitis, liver disorders (Farombi, Adepoju, Ola‐Davies, & Emerole, 2005), the fact that consumption of whole seeds especially for long time produce stimulant‐like effect could induce some toxicological effects which is worth investigating. This study therefore investigates the effects of GK seed on survival, locomotion, and oxidative stress markers in *D. melanogaster*.

## 2 MATERIALS AND METHODS

### 2.1 Materials

#### 2.1.1 Sample collection

Bitter kola (*Garcinia kola* [GK]) fresh seeds were sourced from the Town market in Akure, South‐West, Nigeria and identified at the Crop Soil and Pest Management Department of Federal University of Technology, Akure, Nigeria. The samples were thoroughly washed with running tap water. Thereafter, the seeds were diced into small pieces using a table knife and then air‐dried under shade to obtain a constant weight. Subsequently, the samples were powdered using a Marlex blender. The powdered samples were stored in air‐tight containers and kept in the refrigerator for further analysis.

#### 2.1.2 *Drosophila melanogaster* stock culture

Wild‐type fruit fly (Harwich strain) stock culture (originally from the National Species Stock Centre (Bowling Green, OH, USA) was obtained from Drosophila Laboratory, Department of Biochemistry, University of Ibadan, Oyo State, Nigeria. The flies were maintained and reared on normal diet made up of corn meal medium containing 1% w/v brewer's yeast and 0.08% v/w nipagin at room temperature under 12‐hr dark/light cycle conditions in the Drosophila Research Laboratory, Functional Foods and Nutraceutical Unit, Federal University of Technology, Akure, Nigeria. All the experiments were carried out with the same *D. melanogaster* strain.

#### 2.1.3 Reagents

Chemical reagents such as acetylthiocholine iodide, sulphanilamide, reduced glutathione, n‐n‐diethyl‐para‐phenylenediamine (DEPPD), ferrous sulfate, semicarbazide were procured from Sigma‐Aldrich Co. (St Louis, Missouri, USA). Trichloroacetic acid (TCA) and sodium acetate was sourced from Sigma‐Aldrich, Chemie GmbH (Steinheim, Germany), hydrogen peroxide, methanol, acetic acid, hydrochloric acid, aluminum chloride, potassium acetate, sodium dodecyl sulfate, iron (II) sulfate, potassium ferrycyanide, and ferric chloride were sourced from BDH Chemicals Ltd. (Poole, England). Ascorbic acid and starch were products of Merck (Darmstadt, Germany). Except stated otherwise, all other chemicals and reagents were of analytical grades and the water was glass distilled.

### 2.2 Methods

#### 2.2.1 Experimental design

The flies (both gender, 3–5 days old) were divided into four groups containing 60 flies each. Group I was placed on normal diet alone while groups II–IV were placed on basal diet containing; GK seed at 0.1, 0.5 and 1.0% of diet (equivalent weight replacement) as shown thus;

**Table:** 

Groups	
I	Basal diet
II	Basal diet + 0.1% Garcinia kola (GK) seed
III	Basal diet + 0.5% GK seed
IV	Basal diet + 1.0% GK seed

The flies were exposed to these treatments for 5 days, and the vials containing flies were maintained at room temperature. All experiments were carried out in triplicate (each experimental group was carried out in five independent vials).

#### 2.2.2 Survival study

A study was conducted to assess the effect of dietary inclusion of GK seed on survival rate of flies after 5 days of exposure. Flies (both gender, 3–5 days old) were divided into four groups containing 60 flies each. Each group was exposed to different dietary inclusions of *GK* seed (0.1%, 0.5%, and 1.0%). The flies were observed daily for the incidence of mortality, and the survival rate was determined by counting the number of dead flies for the first 5 days. The data were subsequently analyzed and plotted as cumulative mortality and percentage survival after the treatment period (Abolaji et al., 2014; Adedara, Abolaji, Rocha, & Farombi, 2016).

#### 2.2.3 Measurement of locomotor performance (negative geotaxis)

The negative geotaxis assay was used to evaluate the locomotor performance of flies (Le Bourg & Lints, 1992). In brief, after the treatment period of 5 days, the flies from each group were briefly immobilized in ice and transferred into a clean tube (11 cm in length 3.5 cm in diameter) labeled accordingly. The flies were initially allowed to recover from immobilization for 10 min and thereafter were tapped at the bottom of the tubes. Observations were made for total number flies that crossed the 6‐cm line within a period of 6 s and recorded. The results are expressed as percentage of flies that escaped beyond a minimum distance of 6 cm in 6 s during three independent experiments.

#### 2.2.4 Preparation of tissue homogenate

The flies were immobilized in ice and homogenized in 0.1 M phosphate buffer, pH 7.4. The resulting homogenates were centrifuged at 10,000 × *g*, at 4°C for 10 min in a Kenxin refrigerated centrifuge Model KX3400C (KENXIN Intl. Co., Hong Kong). Subsequently, the supernatant was separated from the pellet into labeled Eppendorf tubes and used for the various biochemical assays.

### 2.3 Biochemical assays

#### 2.3.1 Reactive oxygen species (ROS) level

Reactive oxygen species level in the whole fly tissue homogenates was estimated as H_2_O_2_ equivalent according to the method of Hayashi et al. (2007), with slight modifications. In brief, 50 μl of tissue homogenate and 1,400 μl sodium acetate buffer were transferred to a cuvette. After then, 1,000 μl of reagent mixture of n‐n‐diethyl‐para‐phenylenediamine (DEPPD) (6 mg/ml of DEPPD with 4.37 μM of ferrous sulfate dissolve in 0.1 M sodium acetate pH 4.8) was added at 37°C incubated for 5 min. The absorbance was measured at 505 nm using a spectrophotometer. ROS levels was estimated from an H_2_O_2_ standard calibration curve and expressed as unit/mg protein, where 1 unit = 1 mg H_2_0_2_/L.

#### 2.3.2 Determination of catalase (CAT) activity

Catalase activity in the homogenate samples was determined according to the method of Shina (1972). In brief, 0.1 ml of each tissue homogenate sample was reacted with 0.4 ml 2 M H_2_O_2_ in the presence of 1.0 ml 0.01 M phosphate buffer (pH 7.0). The reaction was stopped by the addition of 2.0 ml dichromate acetic acid. The absorbance of the reaction mixture was taken at 620 nm in a spectrophotometer. A standard curve was prepared by reacting 0.4 mol of 2 M H_2_O_2_ with 2 ml dichromate acetic acid in the presence of 1.0 ml 0.01 M sodium phosphate buffer (pH 7.0). The catalase activity was thereafter calculated and expressed as unit/mg protein where 1 unit = 1 µmol of H202 consumed per minute.

#### 2.3.3 Determination of glutathione‐S‐transferase activity

This assay was carried out according to the method of Habig and Jakoby (1981). It involves the preincubation of reaction mixture containing 1.0 ml 100 mM phosphate buffer (pH 6.5), 30 mM 1‐chloro‐2,4‐dinitrobenzene (CDNB), and 0.7 ml 0f distilled water for 5 mins at 37°C. The reaction was started by the addition of 0.1 ml of the tissue homogenate and 0.1 ml 30 mM glutathione as substrate. The absorbance of the reaction mixture was monitored after 5 min at 340 nm in a spectrophotometer. Reaction mixture without enzyme was used as a blank. The activity of GST was calculated and expressed as unit of GST activity per mg protein.

#### 2.3.4 Determination of the total thiol content

Determination of the level of total thiol content in tissue homogenate was performed by the method of Ellman et al. (1959). The reaction mixture was made up of 270 μl of 0.1 M potassium phosphate buffer (pH 7.4), 20 μl of homogenate, and 10 μl of 10 mM DTNB. This was followed by 30‐min incubation at room temperature, and the absorbance was measured at 412 nm. The total‐thiol content was subsequently calculated and expressed as μmol/mg protein.

#### 2.3.5 Determination of nonprotein thiol content

Determination of nonprotein thiols (NPSH) content was carried out by the method of Ellman (1959). Aliquot amount of Drosophila homogenate was equally mixed with 10% trichloroacetic acid. This allowed for protein precipitation, which was centrifuged at 10,000 × *g* for 5 min at 4°C and the free sulfhydryl groups were determined in the supernatant. The reaction mixture consisting 50 μl of sample, 450 μl phosphate buffer, and 1.5 ml of 0.1 mM of 5,5‐dithiobis 2‐nitro benzoic acid was incubated for 10 min at 37°C. The absorbance was measured at 412 nm, and NPSH level was expressed as μmol/mg of protein.

#### 2.3.6 Measurement of nitric oxide

Nitric oxide (NO) content in the whole fly tissue homogenate was estimated in a medium containing 400 ml of 2% vanadium chloride (VCl_3_) in 5% HCl, 200 ml of 0.1% N‐(l‐naphthyl) ethylene‐diaminedihydrochloride, 200 ml of 2% sulfanilamide (in 5% HCl). After incubating at 37°C for 60 min, nitrite levels, which corresponds to an estimated of level of NO, were determined spectrophotomerically at 540 nm. (Miranda, Espay, & Wink, 2001). Nitrite and nitrate levels were calculated and expressed as nanomole of NO/milligram of protein.

#### 2.3.7 Acetylcholinesterase (AChE) activity assay

Acetylcholinesterase activity was assayed according to the method of Ellman et al. (1961). The reaction mixture was made up of 195 μl of distilled water, 20 μl of 60 mM potassium phosphate buffer (pH 8.0), 20 μl of 20 mM DTNB, 5 μl of homogenate, and 20 μl of 20 mM acetylthiocholine (as initiator). Thereafter, reaction was monitored for 3 min (30‐s intervals) at 412 nm. The AChE activity was thereafter calculated and expressed as mmolAcSch/h/mg protein.

#### 2.3.8 Determination of total protein

Total protein content of fly homogenates was measured by the Coomassie blue method according to Bradford (1976) using bovine serum albumin (BSA) as standard.

### 2.4 Phytochemical screening

#### 2.4.1 Determination of saponin

Saponin was quantified as previously reported by Brunner (1994). This involved 2 g of pulverized sample added to 100 ml of Isobutyl alcohol. The reaction mixture was shaking for 5 hr to ensure uniform mixing using orbital shaker. The reaction mixture was filtered with No 1 Whatman filter paper into a clean beaker, and 20 ml of 40% saturated solution of MgCO_3_ was added. This was followed by another filtration to obtain a clean filtrate. Subsequently, 2 ml of 5% FeCl_3_ was added to 1 ml of the filtrate and made up with distilled water. The reaction was allowed to stand for 30 min for the color development and the absorbance against the blank was taken at 380 nm. Saponin content was expressed in mg/g of sample.

#### 2.4.2 Determination of alkaloid

Alkaloid extract of samples was prepared according to the method of Harborne (1998), with slight modifications (Ademiluyi, Ogunsuyi, Oboh, & Agbebi, 2016). Briefly, 100 g pulverized sample was defatted with n‐hexane for 24 hr. The defatted samples were extracted with 200 ml of 10% alcoholic acetic acid. The mixtures were thereafter filtered first using muslin cloth and then filter paper (Whatman No. 1) to obtain a clear filtrate which was concentrated under vacuum using rotary evaporator (Laborota 4000 Efficient, Heidolph, Germany) at 45°C. Concentrated ammonium hydroxide was subsequently added dropwise to the concentrated filtrate until the precipitate was completed. The whole solution was allowed to settle, and the precipitate was collected and rinsed with dilute ammonium hydroxide to obtain the alkaloid extracts. Alkaloid extract was expressed as mg/g.

#### 2.4.3 Preparation of aqueous extracts

Ten grams of the powdered samples was weighed and extracted with 50 ml distilled water in extraction bottle and shaken vigorously for 2 hr. The extract was filtered using Muslin cloth and further filtered with Whatman filter paper and further centrifuged to obtain clear supernatant.

#### 2.4.4 Determination of cardiac glycosides

Cardiac glycosides were determined as previously reported (Sofowora, 1995). An aliquot of the extract (10 ml) was added in a reaction flask to 50 ml chloroform, which vortexed for 1 hr. The mixture was thereafter filtered followed by addition of 10 ml pyridine and 2 ml of 29% sodium nitroprusside which was shaken thoroughly for 10 min. This was followed by addition of 3 ml of 20% NaOH to develop a brownish yellow color. Digitoxin was used as standard glycoside. The absorbance was read @ 510 nm. The glycoside content was expressed as mg/g of sample.

#### 2.4.5 Determination of total phenol content

The total phenol content of GK seed was determined according to the method of Singleton, Orthofor, and Lamuela‐Raventos (1999) with some modifications (Oboh & Ademosun, 2012). Briefly, the reaction mixture consists of an aliquot of the extract, 2.5 ml 10% Folin‐ciocalteau's reagent (v/v), and 2.0 ml of 7.5% Na_2_CO_3_. This was followed by incubation for 40 min at 45°C, and the absorbance was quantified at 765 nm in a spectrophotometer. The total phenol content was subsequently calculated as mg/g gallic acid equivalent.

#### 2.4.6 Determination of total flavonoid content

The total flavonoid contents of Gk seed was determined using the modified method of Meda, Lamien, Romito, Millogo, and Nacoulma (2005) as reported by Oboh and Ademosun (2012). Briefly, the reaction mixture consists of 0.5 ml of extract, 0.5 ml of methanol, 50 μl 10% aluminum chloride, 50 μl 1 M Potassium acetate, and 1.4 ml distilled water. The reaction was incubated at room temperature for 30 min. This was followed by absorbance quantification at 415 nm. The total flavonoid content was subsequently calculated and expressed as mg/g quercetin equivalent.

### 2.5 Data analysis

The results of replicate readings were pooled and expressed as mean ± standard deviation (*SD*). One‐way analysis of variance (ANOVA) was used to analyze the results followed by Bonferoni's post hoc test, with levels of significance accepted at *p* < 0.05, *p* < 0.01, and *p* < 0.001. All statistical analysis was carried out using the software Graph pad PRISM (V.5.0).

## 3 RESULT

**Figure 1:**
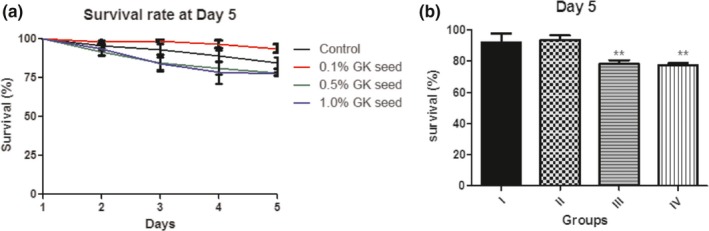
(a) Survival rate (%) of *Drosophila melanogaster* fed diet supplemented with *Garcina kola* (GK) seed for 5 days. (b) day 5 survival rate (%) of *D. melanogaster* fed diet supplemented with *Garcina kola* (GK) Seed. Values represent mean ± *SD*. Groups: I: Normal diet; II: Normal diet + 0.1% *Garcinia kola* (GK) seed; III: Normal diet + 0.5% GK seed; IV: Normal diet + 1.0% GK seed

The effect of dietary inclusions of *Garcinia kola* (GK) seed (0.1%, 0.5% and 1.0%) on 5‐day survival of *D. melanogaster* (Figure 1a and b) was assessed. This revealed that there were 14.9% and 16.4% reduction in survival observed in flies fed with 0.5% and 1.0% dietary inclusions of GK seed, respectively, compared to control. Interestingly, 0.1% inclusion of GK seed increased flies survival by 8.37% at day 5, against the control group.

The effect of dietary inclusions of *Garcinia kola* (GK) seed (0.1%, 0.5% and 1.0%) on 5‐day survival of *D. melanogaster* (Figure 1a and b) was assessed. This revealed that there were 14.9% and 16.4% reduction in survival observed in flies fed with 0.5% and 1.0% dietary inclusions of GK seed, respectively, compared to control. Interestingly, 0.1% inclusion of GK seed increased flies survival by 8.37% at day 5, against the control group.

**Figure 2:**
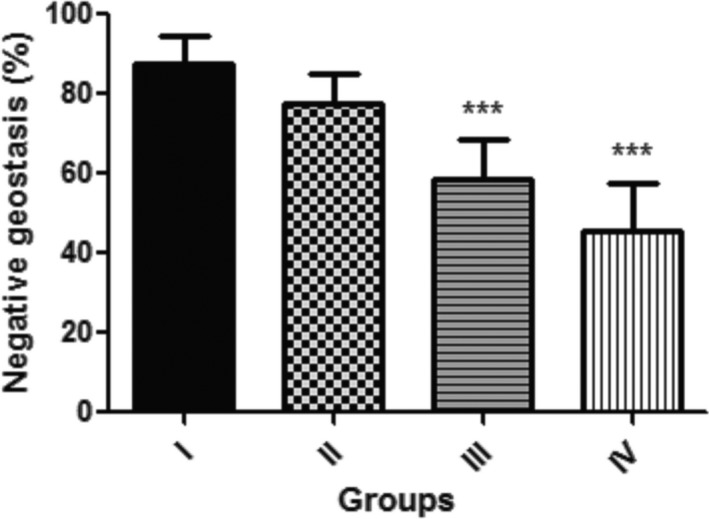
Percentage locomotor performance (Negative Geostasis) of *Drosophila melanogaster* fed diet supplemented with *Garcina kola* (GK) Seed. Values represent mean ± *SD*. ***Values are significantly different at *p* < 0.001. Key: as described for Figure 1

Figure 2 reveals the locomotor performance of flies fed with GK seed supplemented diet (0.1%, 0.5% and 1.0%). This revealed that dietary inclusion of 0.5% and 1.0% GK seed decrease significantly (*p* < 0.001) the locomotor performance of the flies in contrast to the control, while there is no significant difference (*p* > 0.05) between locomotor performance in control and flies fed with 0.1% GK seed supplemented diet.

**Figure 3:**
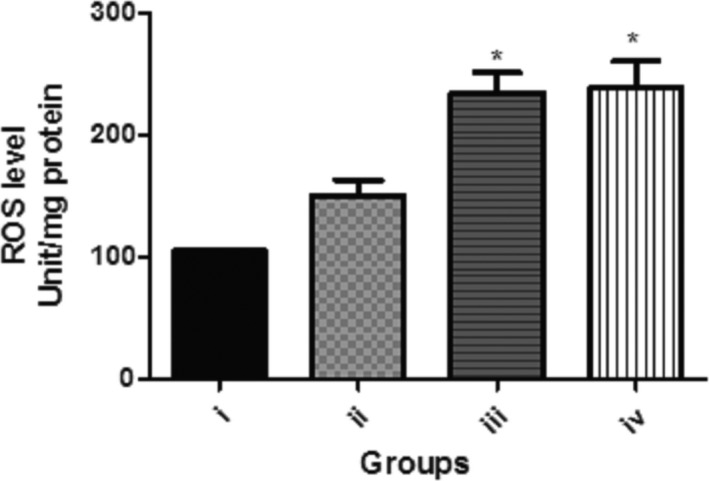
Effect of dietary inclusions of *Garcina kola* (GK) seed on ROS level in *Drosophila melanogaster*. Values represent mean ± *SD*. *Values are significantly different at *p* < 0.05. Key: as described for Figure 1

**Figure 4:**
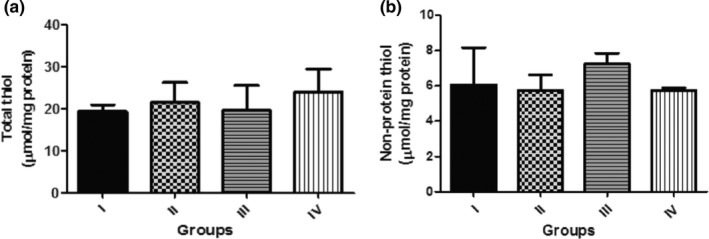
(a) Effect of Dietary Inclusions of *Garcina kola* (GK) seed on total thiol content in *Drosophila melanogaster*. (b) Effect of dietary inclusions of *Garcina kola* (GK) seed on nontotal thiol content in *D. melanogaster*. Values represent mean ± *SD*. Key: as described for Figure 1

Figure 3 shows the effect of dietary inclusion of GK seed (0.1%, 0.5% and 1.0%) on ROS level in *D. melanogaster*. It was shown that there was significant (*p* < 0.05) increase in ROS level in the groups treated with 0.5% and 1.0% GK seed. Also, Figure 4a and b shows the effect of dietary inclusion of GK seed (0.1%, 0.5%, and 1.0%) on total thiol and nonprotein thiol contents in *D. melanogaster*. These results showed no significant difference (*p* > 0.05) in total thiol and nonprotein thiol contents among the various treatment groups.

**Figure 5:**
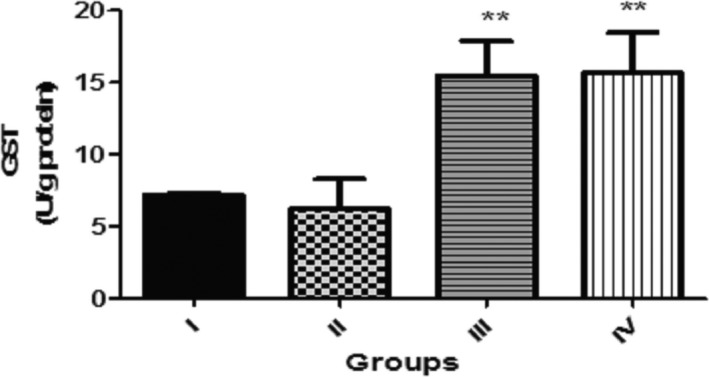
Effect of dietary inclusions of *Garcina kola* (GK) seed on glutathione‐S‐transferase (GST) activity in *Drosophila melanogaster*. Values represent mean ± *SD*. **Values are significantly different at *p* < 0.01. Key: as described for Figure 1

Figure 5 presents the effect of dietary inclusion of GK seed (0.1%, 0.5%, and 1.0%) on glutathione‐S‐transferase (GST) activity in *D. melanogaster*. This showed that flies given diet with 0.5% and 1.0% GK seed exhibited significantly higher (*p* < 0.01) GST activity against the control. However, GST activity in flies fed with 0.1% GK seed showed no significant different (*p* > 0.05) compared to the control.

**Figure 6:**
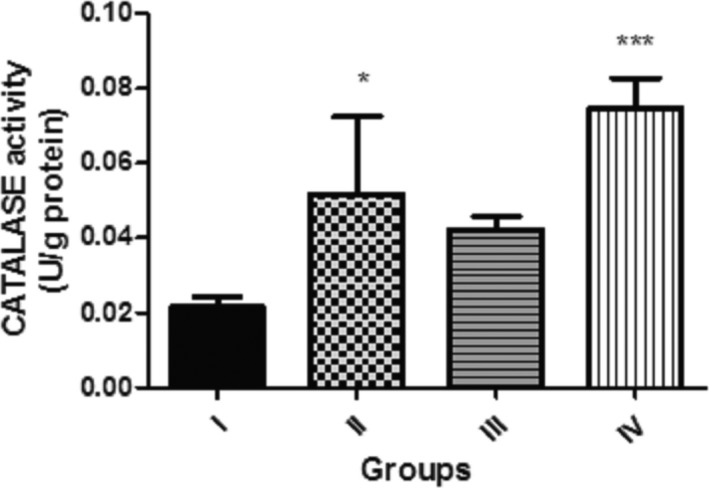
Effect of dietary inclusions of *Garcina kola* (GK) seed on catalase activity in *Drosophila melanogaster*. Values represent mean ± *SD*. *Values are significantly different at *p* < 0.05; ***Values are significantly different at *p* < 0.001. Key: as described for Figure 1

Presented in Figure 6 is the effect of dietary inclusion of GK seed (0.1%, 0.5%, and 1.0%) on catalase activity in *D. melanogaster*. This showed that flies fed with 0.1% and 1.0% GK seed supplemented diet exhibited significantly higher (*p* < 0.001 and *p* < 0.05) catalase activity against the control. However, catalase activity in flies fed with 0.5% GK seed was not significantly different (*p* > 0.05) against the control.

**Figure 7:**
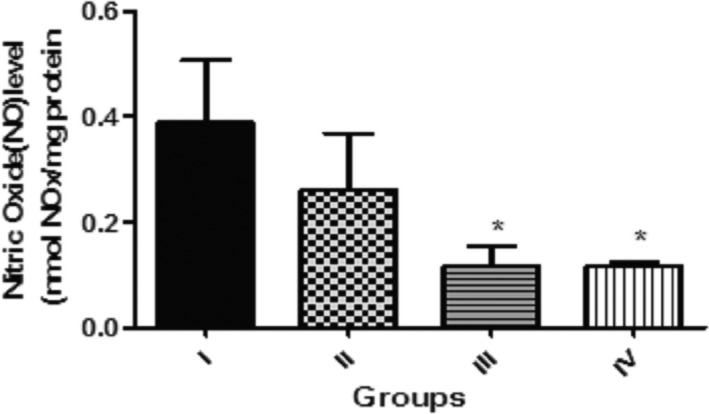
Effect of dietary inclusions of *Garcina kola* (GK) seed on nitric oxide (NO) content in *Drosophila melanogaster*. Values represent mean ± *SD*. * Values are significantly different at *p* < 0.05. Key: as described for Figure 1

In Figure 7, the effect of dietary inclusions of GK seed (0.1%, 0.5%, and 1.0%) on nitric oxide (NO) content in *D. Melanogaster* was presented. This showed that flies given diet with 0.5 and 1.0% GK seed significantly lowered (*p *< 0.05) the NO content against the control. However, NO content in flies raised on diet supplemented with 0.1% GK seed shows no significant different (*p* > 0.05) against the control.

**Figure 8:**
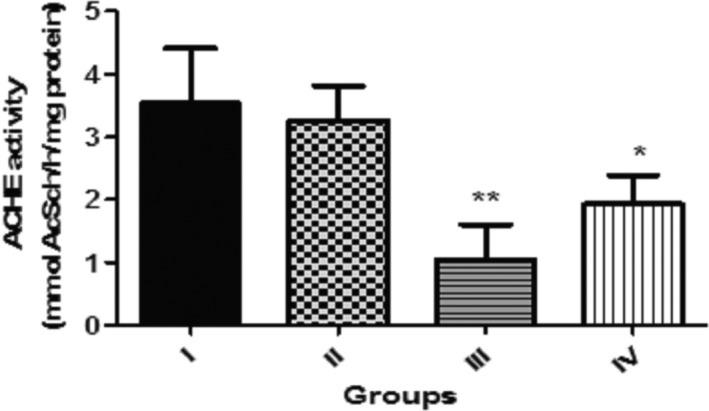
Effect of dietary inclusions of *Garcina kola* (GK) seed on acetylcholinesterase (AChE) activity in *Drosophila melanogaster*. Values represent mean ± *SD*. *Values are significantly different at *p* < 0.05; **Values are significantly different at *p* < 0.01. Key: as described for Figure 1

Figure 8 shows the effect of dietary inclusion of GK seed (0.1%, 0.5% and 1.0%) on acetylcholinesterase (AChE) activity in *D. melanogaster*. This reveal that flies given diet with 0.5 and 1.0% GK seed exhibited significantly lower (*p* < 0.01, *p* < 0.05) AChE activity against the control. However, AChE activity in flies given diet with 0.1% GK seed was not significantly different (*p* > 0.05) against the control.

Table 1 presents the Pearson correlation coefficient (*r*) vales between the survival rate of treated flies across the groups and the studied bioassays. This showed that there was a strong correlation especially among flies’ survival rate, the ROS level, and the AChE activity; Table 2 presents the phytochemical screening of GK seed. This showed that GK seed has higher abundance of saponin (673.41 ± 10.67 mg/g) than other phytochemicals such as glycosides (218.65 ± 11.37 mg/g), alkaloid (21.2 ± 6.22 mg/g), phenols (2.09 ± 0.03 mg/g), and flavonoids (0.08 ± 0.02 mg/g).

**Table 1:** Pearson's correlation coefficient (*r*) between flies’ survival rate and selected bioassays

Groups	ROS	AChE	NO	Total Thiol	Nonprotein Thiol	GST	Catalase
I	0.886	0.756	−0.858	−0.331	−0.529	0.750	0.885
II	0.799	0.692	0.571	0.328	0.624	0.685	0.710
III	−0.553	0.996	−0.333	0.887	−0.333	−0.529	−0.522
IV	0.946	−0.550	−0.550	0.115	0.579	−0.447	0.808

*Note*

Groups: I: Basal diet; II: Basal diet + 0.1% Garcinia kola (GK) seed; III: Basal diet + 0.5% GK seed; IV: Basal diet + 1.0% GK seed.

**Table 2:** Phytochemical screening of *Garcina kola* (GK) seed

Phytochemicals	mg/g
Saponin	673.41 ± 10.67
Glycoside	218.65 ± 11.37
Alkaloid	21.2 ± 6.22
Total phenol	2.09 ± 0.03
Total flavonoid	0.08 ± 0.02

*Note*

Values represent mean ± *SD*.

## 4 DISCUSSION

Results from this study revealed that higher (0.5% and 1.0%) dietary inclusions of GK significantly reduced fly locomotion after just 5 days of exposure. This suggests that the seed induced some toxicological effects at these concentrations. These observations correlate well with their reduction in survival rate during the 5‐day treatment; with 14.9% and 16.4% reduction in survival observed in flies fed 0.5% and 1.0% dietary inclusions of GK, respectively, compared to control. Conversely, dietary inclusion of 0.1% GK seed caused 8.9% increase in flies’ survival and produced no significant difference in the locomotor performance compared to control. These suggest that GK seeds could offer some medicinal properties at lower concentration and toxicological effects at higher concentrations.

The oxidative stress hypothesis of aging has been well reported in various animal models (Forbes, Coughlan, & Cooper, 2008; Negre‐Salvayre, Salvayre, Auge, Pamplona, & Portero‐Otin, 2009). Specifically, there have been several reports implicating ROS generation and oxidative stress in reduced life span in *D. melanogaster* (Hyrsl, Büyükgüzel, & Büyükgüzel, 2007; Lozinsky, Lushchak, Storey, Storey, & Lushchak, 2012; Lozinsky et al., 2013). Therefore, this study investigated the possibility of ROS generation and oxidative stress as possible mechanisms behind the reduction in survival rate of Drosophila fed GK seed at higher percentage inclusions. It was observed that dietary inclusions of GK seeds at higher percentage inclusions caused significant elevation in ROS production in the flies. Interestingly, the Pearson correlation analysis revealed that there was strong correlation between treated flies’ survival rate and ROS level with *r* = 0.946 for flies treated with 1.0% GK seed. This showed that elevation in ROS level would have contributed to the decline in survival rate of flies experienced at higher (0.5% and 1.0%) GK seed inclusions.

Dietary inclusions of GK seed (0.5% and 1.0%) increased significantly the activities of Catalase and Glutathione S‐transferase (GST). Indeed, cellular macromolecules are protected primarily from the insult of free radical species by endogenous antioxidant molecules including catalase, glutathione peroxidase, glutathione‐S‐transferase, and superoxide dismutase (Rand, 2010). Catalase catalyzes the reduction of H_2_O_2_ to H_2_O and O_2_ and thus protects biological tissues from the deleterious peroxidative effect of H_2_O_2_ (Abolaji, Olaiya, Oluwadahunsi, & Farombi, 2017). Therefore, the increase in these antioxidant enzymes could be associated with induction of ROS production in the flies by GK seeds inclusions.

The elevated ROS level in the flies signifies a state of redox imbalance and oxidative stress. This could explain the elevation in these antioxidant enzymes as an adaptive response to the oxidative assaults. Adaptive response, which is the ability of an organism to effectively counteract cellular damages induced by cytotoxic agents such as free radicals, has been identified in *D. melanogaster* (Abolaji et al., 2017). Such elevation in antioxidant enzymes’ activities as an adaptive response to cytotoxic agents has been previously reported in *D. melanogaster,* and they are often accompanied by impairments in cellular thiol levels (Abolaji et al., 2017; Bayliak et al., 2018; Perkhulyn et al., 2017). Thiols represent the major portion of cellular antioxidant systems, and they play a vital role in defense against ROS (Mungli, Shetty, Tilak, & Anwar, 2009). However, the overall response of thiols to oxidative cellular assaults is often a product of interactions between thiol oxidation and synthesis (Osburn et al., 2006). Therefore, the nonsignificant difference in thiol contents among the treatment groups in this study could be as a result of correlation between thiol consumption in response to the presence of ROS and replenishing as a result of adaptive response under acute exposure.

Furthermore, investigations into the basis of the reduction in survival rate of flies fed with the dietary inclusions were necessitated. Acetylcholinesterase (AChE) hydrolyses the neurotransmitter‐acetylcholine that regulates locomotion and motor function (Day, Damsma, & Fibiger, 1991). Higher dietary inclusions of GK seeds (0.5% and 1.0%) showed a significant decrease in survival rate also induced a decrease in AChE activity. The decrease in the AChE activity following dietary inclusions of GK seeds at 0.5% and 1.0% could be an increase in acetylcholine levels in the synaptic cleft and as a result induce cholinergic toxicity which could impair neuromuscular activities such as climbing abilities of flies (Akinyemi, Oboh, Ogunsuyi, Abolaji, & Udofia, 2017). It should also be noted that prolonged reduction in the activity of AChE in the flies could lead to oxidative stress which could also contribute to their reduced survival rate (Olney, Collins, & Sloviter, 1986). This is further corroborated by the strong correlation between the survival rate of flies and their AChE activities among the treated groups.

Nitric oxide is a diffusible signaling molecule, and its presence in both vertebrates and insects nervous system has been established (Müller, 1997). In *D. melanogaster*, NO has been well reported in cellular development during the developmental stages of the flies (Enikolopov, Banerji, & Kuzin, 1999; Jaszczak, Wolpe, Dao, & Halme, 2015). Studies have also shown that NO function in immune responses of the flies to pathogens and parasites (Eleftherianos et al., 2014; Nappi, Vass, Frey, & Carton, 2000). Although there is dearth of information on the role of NO in adult fly physiology, nevertheless, the production of NO is reportedly more confined to the brain of adult flies (Enikolopov et al., 1999). It was shown in this study that dietary inclusions of GK seeds at 0.5% and 1.0% showed a significant decrease in endogenous NO contents in the flies. This significant reduction in NO content could impair its signaling role. However, at this level, whether or not this observation contributes to the overall reduction in survival rate and locomotor performance of flies fed with 0.5% and 1.0% dietary inclusions of GK seed might not be completely understood and hence deserves further studies. Nevertheless, in mammalian cells; NO has protective role as antioxidants and regulatory role as vascular tone and cell signaling modulator at physiological concentrations, while at elevated levels, it could elicit deleterious effects by reacting with superoxides to form reactive nitrogen oxide species that can induce oxidative damages to macromolecules (Smith, Kapoor, & Felts, 1999; Wink et al., 2001). NO's protective roles on mammalian cells are associated with such mechanisms as scavenging radical species, formation of iron–nitrosyl complexes that limits availability of iron as pro‐oxidant, prevention of lipid peroxidation reactions, attenuation of enzymatic (xanthine oxidase), and nonenzymatic (H_2_O_2_)‐mediated cell death, as well as up‐regulating antioxidant enzymes’ activities (Hasanuzzaman et al., 2017; Park, 1996; Wink et al., 2001). These observations suggest that a decrease in NO production may elicit oxidative responses that could culminate in increase ROS level and altered antioxidant enzyme activities.

Natural toxicants present in human foods and animal feeds present a potential hazard to health. Saponins are phytochemicals found in variety of plant foods. They exhibit strong foaming ability in aqueous solutions, as well as cytotocyic effect (Haridas, Arntzen, & Gutterman, 2001; Milgate & Roberts, 1995), they also demonstrate hemolytic properties (Milgate & Roberts, 1995; Oakenfull & Sidhu, 1989; Takechi, Doi, & Wakayama, 2003) which also inhibits the activities of proteases (Wierenga & Hollingworth, 1992). Saponins have been reported to be toxic to humans and pests (Chaieb, 2010). A study also reported the increase in mortality rate of L5 *Schistocerca gregaria* larva insect when injected with crude saponins extracted from *Cestrum parqui* (Barbouche, Hajem, Lognay, & Ammar, 2001). Furthermore, *Leptinotarsa decemlineata* larvae were reported to be toxic to saponins extracted from the leaves and the roots of alfalfa (Szczepanik, Krystkowiak, Jurzysta, & Bialy, 2001). Another study reported the larvicidal activity of saponins extracted from *Quillaja saponaria* against the mosquitos’ larvae of two species *Aedes aegypti* and *Culex pipiens*; their results showed 100% mortality using 1,000 mg/L during 5 days (Ikbal, Monia, & Habib, 2007; Pelah, Abramovich, Markus, & Wiesman, 2002). Crude saponins of *Cestrum parqui* also showed toxicity on some insects such as (*Schistocera gregaria, S. littoralis,* and *Tribolium confusum*); in all the toxicity study reported, the larvae of the mosquito *Culex pipiens* was the most significant toxicity was observed (Chaieb, 2005; Milgate & Roberts, 1995). Consequently, the observed high abundance of saponin in GK seed could be one of the major reasons for the mortality and impairment in locomotor performance in flies. Also high abundance of glycosides, which have been reported to be neurotoxic and cardiotoxic (David et al., 2013), could also contribute to the impairment in locomotor performance.

## 5 CONCLUSION

The results showed that dietary inclusions of GK seed especially at high percentage reduced survival rate and locomotor performance of the flies. It also produced some cholinergic impairment and increased ROS production. These observations could be attributed to the predominant phytochemicals in GK seed‐saponins and glycosides that have been reported to be toxic at high concentration. However, dietary inclusion of 0.1% of GK seed seems tolerable to flies and even produced some percentage increase in flies’ survival rate, no significant effect on locomotor performance, and nonimpairment in AChE activity compared to the control. This study therefore suggests that high consumption of GK seed in *D. melanogaster* induces some toxicological effect, while moderate–low consumption could be beneficiary and offer the well reported medicinal effects attributed to GK seeds.

## CONFLICT OF INTEREST

The authors declare that they do not have any conflict of interest.

## ETHICAL STATEMENT

This study does not involve any human or animal testing that requires ethical approval.
